# Turner syndrome transition clinic in the West of Scotland: a perspective

**DOI:** 10.3389/fendo.2023.1233723

**Published:** 2023-09-01

**Authors:** Baryab Zahra, Aparna Sastry, Marie Freel, Malcolm Donaldson, Avril Mason

**Affiliations:** ^1^ Department of Paediatric Endocrinology, Queen Elizabeth University Hospital, Glasgow, United Kingdom; ^2^ Assisted Conception Service, Glasgow Royal Infirmary, Glasgow, United Kingdom; ^3^ Department of Endocrinology, Queen Elizabeth University Hospital, Glasgow, United Kingdom; ^4^ Section of Child Health, Glasgow University School of Medicine, Glasgow, United Kingdom

**Keywords:** Turner syndrome, cardiovascular, HRT, blood pressure, transition

## Abstract

**Introduction:**

Turner Syndrome (TS) is the commonest chromosomal abnormality in females. Establishing and maintaining long-term follow-up after transition to adult endocrine services, to allow for essential lifelong surveillance of hypertension and cardiovascular disease, and optimal hormone replacement, remains a challenge. A TS transition clinic was established with the aim of supporting successful transfer and establishing long-term follow-up in adult endocrine services. Our objectives are to evaluate the success of our TS transition service primarily in achieving and maintaining follow-up after transfer to adult services and to assess the adequacy of health surveillance post-transition with a specific focus on cardiac monitoring and hormone replacement.

**Methods:**

A departmental database was used to identify young people whose care had transferred to adult endocrine services. An electronic case record was utilised to obtain clinic attendance and relevant clinical information on cardiovascular monitoring and hormone replacement therapy (HRT).

**Results:**

Forty-six (n=46) young people transferred to adult endocrine services during the observed 20-year period, 1998-2017. Thirty-six (n=36) had transferred prior to 2015, of whom sixteen (n=16, 44%) are lost to long-term follow-up at 5 years. Overall, 41 (89%) patients have had cardiac imaging surveillance since transferring, However, only 30 (73%) of these were carried out at the recommended frequencies. All 20 women in established follow-up have had cardiac imaging. Five out of the 46 (11%) patients do not have any documented cardiovascular monitoring. Forty (86.9%) women have had a documented BP measurement. Nineteen of the 20 women who are in 5- year established follow-up have a documented blood pressure. Five (11%) women are not on HRT, while two (4%) remain on oestrogen-only HRT. Thirty-seven (80.4%) women are on combined HRT, only eight (21.6%) are on the recommended form of oestradiol. Two (4%) are not on HRT due to normal ovarian function.

**Conclusion:**

A significant proportion of girls with TS are currently lost to adult endocrine services. Strategies to improve long-term endocrine follow-up are needed to ensure lifelong health needs and adequate hormone replacement are met. Whilst similar parameters are monitored in adult endocrine services a group of patients may be at risk of receiving inadequate HRT and developing cardiovascular complications.

## Introduction

Turner Syndrome affects 1/2500 of live female births and is the commonest chromosomal abnormality in females (1). TS continues to be diagnosed across the lifespan with peaks during foetal life, infancy, late pre-pubescence (8–12years) and during late adolescence/early adulthood (2). The first presentation to healthcare services is often consequential to short stature or pubertal delay, however, the clinical presentation of TS ranges from a classic appearance with many physical differences to individuals who have no apparent or minimal observable features (1).

Optimal care in TS includes regular monitoring through childhood, adolescence and into adult life to screen for associated complications. In childhood and adolescence early screening and intervention are required to offset the following problems: short stature, delayed puberty, abnormal alignment of one or both eyes, hearing loss, heart and kidney abnormalities, thyroid dysfunction, and gluten intolerance. In late adolescence and early adulthood, screening and intervention are required to offset the following additional complications: hypertension, coarctation and aortic dissection (3), hypogonadism and infertility, osteoporosis, hearing impairment, increased risk of developing diabetes, and obesity (4).

Young women with TS have complex health needs which require life-long treatment through seamless transfer from paediatric to adult services. Management of any individual with TS requires support from an extensive and non-exhaustive list of specialties which may include the following: endocrinology, cardiology, gynaecology, audiology, ENT, urology, renal medicine, and clinical psychology. Establishing and maintaining long-term follow-up after transition to adult services remains a challenge to paediatric and adult services and requires active participation from both (5).

A TS transition clinic was set-up in 1998 at the Royal Hospital for Children Glasgow (RHCG), to coordinate transfer from paediatric endocrine to adult endocrine clinics. The TS Transition clinic serves to introduce girls with TS to members of the adult multi-disciplinary endocrine team in a paediatric setting with the aim of supporting a successful transition and establishing long-term follow-up in adult life. The primary aim of our study is to establish the success of the TS transition clinic, using the proportion of girls with TS who remain in established adult endocrine follow-up at three and five years after transfer as the primary outcome measure. Our secondary aim was to establish if the recommendations outlined in European Society for Endocrinology with respect to monitoring for cardiovascular complications (hypertension, aortic dilatation and dissection) and hormone replacement therapy (HRT) were adhered to following transfer to adult services. Recommendations include checklists, these can be used during the process of transitioning to ensure appropriate parameters are monitored. Cardiovascular complications are the commonest cause of early mortality and research has shown a reduced lifespan by 10 years in TS patients (6). Additionally, adequate HRT use has been shown to result in reduced hospitalisations with osteoporotic fractures and strokes (7). We therefore focused on assessing compliance with recommendations for these parameters. The current recommendations for cardiac monitoring suggest performing transthoracic echo (TTE) or Cardiac MRI (CMR) every 5 years in paediatrics and every 10 years in adult services in the absence of significant cardiac disease or abnormality. If cardiac abnormality such as Bicuspid Aortic Valve (BAV) or Coarctation of Aorta (CoA) exists then the recommendation is to perform TTE/CMR every 2-3 years or every 6 to 12 months, respectively. It is suggested to monitor BP annually and treatment should be commenced in a timely manner if indicated in the form of a beta- blocker, angiotensin receptor blocker or both (6). Hormone replacement therapy in Turner Syndrome is important for achieving adequate bone mass and maintaining optimal reproductive function, as well as facilitating development of secondary sexual characteristics. Pubertal induction, in the presence of ovarian insufficiency, should be commenced at the age of 11-12 years with incremental doses of oestradiol building to an adult dose of replacement over 2-3 years. 17 β-oestradiol (E2) is known to be more physiological compared to synthetic forms and is therefore recommended, with transdermal route preferred over the oral route to avoid first pass metabolism (8). A previous study has analysed oestrogen replacement trends in a large population of TS patients (n=627) and shows administration of oral and transdermal E2 increased collectively by 2010 in comparison to use before 1995, reinforcing recommendations of preferential use of E2 as set out by the European Society for Endocribology (9). Progesterone is added once tanner stage 4 is attained or after 2-3 years of unopposed oestrogen or after an initial breakthrough bleed has occurred. Hormone replacement therapy, with oestrogen and progesterone, should be continued until an age of natural menopause is reached (2).

## Materials and methods

A paediatric endocrine departmental database containing information on all TS patients who had attended the TS transition clinic at the Royal Hospital for Children, Glasgow, and had subsequently had their care transferred to an adult endocrine service (between years 1998 and 2017) was used. Information collected from the database included the dates of attendance/non-attendance at the TS transition and adult Endocrine clinics. Relevant clinical data collected from the database included the following: information on co-morbidities, cardiovascular complications (hypertension, bicuspid aortic valve and previous surgery for coarctation), evidence of preserved ovarian function with spontaneous onset of puberty and regular menses, prescribed hormone replacement therapies. Additional clinical data, following transfer to an adult endocrine service, was gathered using access to a clinical electronic medical record. Information sourced from the clinical electronic medical record included the following: secondary/tertiary care clinical appointment letters, blood test results, current active prescriptions, upcoming clinic appointments with dates, previously attended and missed clinical appointments in the West of Scotland. The information collated from the clinical electronic medical record included the following: frequency of blood pressure measurement in an adult clinical setting, values of systolic and diastolic blood pressures were compared to standards used to diagnose hypertension in an adult population, current treatment with an anti- hypertensive therapy, frequency of cardiac imaging in an adult setting (echocardiogram or cardiovascular MRI), available obstetric/pregnancy history and current hormone replacement therapy, oestradiol (E2) and progesterone are currently recommended (8)

## Results

### Follow-up

Overall, 53 girls had attended a Turner transition clinic and transferred to an adult endocrine clinic between 1998 and 2017 inclusive. Seven cases were excluded from further analysis because of: relocation outside the region (n=1), incomplete information (n=5) and deceased (n=1). Attendance was assessed in the remaining 46 girls, median 18.3 years (range, 16.0-21.5) at time of transfer. Of these 46 girls, 36 had their care transferred to an adult endocrine service before 2015 and their data were used to assess long- term follow up in an adult endocrine clinic at three and five years. Of these patients, 26/36 (72.2%) were in established follow-up at three years and 20/36 (55.5%) remained in established follow-up at 5 years.

### Assessment of adequacy of hormone replacement therapy

**Table 1** shows HRT preparation currently in use by 43 women. A combined form of progesterone and oestrogen is in use by 37/46 (80%), however only 8 (21.6%) are on the recommended form of oestradiol. Two out of 46 (4%) are only on one form of hormone replacement (Ethinylestradiol), and 2/46 (4%) are not on any form of replacement at present as deemed to have normal ovarian function and regular menses. Finally, 5/46 (10%) have no record of active prescriptions, all of whom are lost to long-term follow-up.

**Table 1:** Details of different hormone replacement prescriptions given to 46 women with Turner syndrome who were transferred to adult care from 1998-2017.

Num. of Patients	HRT Brand name as prescribed by practitioner	Active Drugs	Method of Administration	Compliant with recommendation
12	Rigevidon	Ethinylestradiol & Levognesterol	Oral	n
7	Gedarel	Ethinylestradiol & Desogestrel	Oral	n
2	Ethinylestradiol	Ethinylestradiol	Oral	n
2	Norethisterone & Estradot Patches	Estradiol & Norethisterone	Transdermal	y
4	Ethinylestradiol & Levognesterol	Ethinylestradiol & Levognesterol	Oral	n
4	Evorel	Estradiol & Norethisterone	Transdermal	y
1	Millinete	Ethinylestradiol & Gestadone	Oral	n
4	Ethinylestrasiol & Norethisterone	Ethinylestrasiol & Norethisterone	Oral	n
1	Novefem	Estradiol & Norethisterone	Oral	y
1	Microgynon	Ethinylestradiol & Levognesterol	Oral	n
1	Femoston	Estradiol & Dydrogesterone	Oral	y
5	Unknown	not applicable	Not Applicable	n
2	Spontaneous pubery	not applicable	Not Applicable	Not Applicable

Both trade and chemical names for each preparation are given. Method of administration either given orally or transdermal patches.

Transdermal preparation is used by 6/39 (15.4%) only, oral preparations are used by thirty-three (84.6%).

### Cardiovascular monitoring

#### Blood pressure

Forty out of 46 women (86.9%) have a documented BP measurement in any tertiary clinics, with sixteen (40%) of these having been monitored in the adult endocrine clinic and twenty-four (60%) monitored in the adult cardiology clinic. Nineteen of the 20 women who are in 5-year established follow-up have a documented blood pressure. The remaining 6 women who have no recorded measurement of blood pressure, of those only one (16%) is in established follow-up. Eight out of 40 women (20%) with a documented BP measurement have a BP in a hypertensive range and are currently on anti-hypertensive treatment.

#### Cardiac imaging with echocardiogram or cardiac MRI

Forty-one out of the 46 women (89%) have had cardiac imaging (Echocardiogram or Cardiac MRI) performed since transfer to adult healthcare services, of which 30 (73%) were performed as per recommendations. All 20 women in established follow-up have had cardiac imaging. Two women out of this cohort who are in established follow-up, have had successful pregnancies with no reported complications, only one had additional cardiac monitoring. **Table 2** outlines any cardiac asnipbnormalities within our patient group and if this meets recommended monitoring standard. The remaining five women who have no documented cardiac imaging following transfer to adult care, only one (20%) is in established follow-up.

**Table 2:** Percentage of patients meeting required cardiac monitoring standards depending on cardiac anatomy in TS.

*Anatomical Abnormality*	*Num. of Patients & Percentage Meeting Recommendations*
*Conarctation of Aorta*	3 (0%)
*BAV*	7 (57%)
*Tricuspid AV with fusion Aortic root dilation*	1 (0%)
	1 (100%)
*Nil*	3 4(70%)

BAV, biscuspid aortic valve; AV, aortic valve.

## Discussion

TS is a complex disorder requiring multi-disciplinary care throughout life and would benefit from seamless transition of medical care between paediatric and adult services. TS patients continue to have a 3-fold higher mortality risk than the general population in all major causes of death (10). Cardiovascular related deaths account for 40% of this excess risk, and therefore, attendance at an appropriate adult clinic beyond paediatric care is essential (11). Following the International Turner Syndrome Meeting in 2016, clear guidance has been issued suggesting optimal screening and timing of cardiovascular imaging during transition and into adult life and should be adopted for those transitioning after this period (2). In addition, regular monitoring of modifiable risk factors for cardiovascular disease, including hypertension, obesity, and metabolic syndrome, are imperative. The health burden imposed by the life- long risk of associated co-morbidities in TS is more difficult to quantify. Recommended surveillance for endocrine complications and other organ complications, with timely intervention, should continue at the recommended frequencies across the lifespan.

In response to international guidance and recommendations outlined by the Endocrine Society a TS transition clinic was initially set-up in 1998 at the Royal Hospital for Children Glasgow. The TS Paediatric and Transition clinic has subsequently co-located with the designated Adult TS clinic since 2015 providing care for girls and women in the West of Scotland at a single site. The process of transition is planned and staged and makes use of TS specific materials including checklists outlining the recommended frequency of investigations and clinical summary documents to aid communication between paediatric and adult teams.

This review of the TS Transition clinic highlights that a substantial number of girls in our clinic are not in established adult endocrine follow-up at three- and five-years following transfer and is higher than previously reported in other centres (12). However, we have observed that most girls not in established adult endocrine follow-up attend clinics in other specialties including Cardiology, Gastroenterology, ENT and Dermatology. We can infer that young women attending clinics other than adult endocrinology have not understood the need for lifelong follow-up in this service. It is not clear if this group of young women, not in regular adult endocrine follow-up whilst attending other specialty clinics, are receiving the necessary monitoring, hormonal replacement and surveillance recommended in adult Turner clinics. The young women who are lost to endocrine follow-up are not in receipt of sufficient oestrogen replacement or cardiovascular monitoring based on data available from a clinical electronic medical record. In addition, there is lack of information on their metabolic parameters such as weight, cholesterol status given the increased risk of cardio-metabolic complications in TS patients these need to be monitored and evaluated in further reviews of this service.

Cardiac monitoring beyond initial identification of congenital cardiac anomalies remains an integral aspect of TS patient care throughout life. The international consensus recommends cardiac imaging (either Transthoracic ECHO or Cardiac MRI) at the time of diagnosis and in the absence of cardiac anomaly to be performed at either 5-year interval in paediatric care and at a 10-year interval in adult care except for pregnant women who are recommended to undergo further imaging. In the presence of hypertension or cardiac anomalies (bicuspid aortic valve and previous coarctation) more frequent monitoring is recommended (2). The women who were in established follow-up were having regular measurements of blood pressure in either adult cardiology or endocrinology clinics and had cardiac imaging performed at recommended intervals based on a pre-determined risk of aortic dissection. Whilst guidance is to have additional cardiac monitoring during pregnancy, this was only the case for one patient.

Amongst the many complications of TS, gonadal failure occurs in almost all patients, resulting in infertility and in most cases absence of puberty (13). In a small number of patients, spontaneous puberty occurs but there is an increased risk of ovarian insufficiency therefore regular monitoring is recommended (14). Similarly, to post-menopausal females, the risk of developing co-morbidities due to lack of oestrogen such as osteoporosis is the same in TS patients with ovarian failure therefore it is recommended to commence hormone replacement therapy from the age of 11 or 12 until age of menopause (2). Except for a small proportion of women who have had previous normal onset of puberty and have regular menses most women in our cohort are on a combined form of hormonal replacement. The recommendation is to use natural oestrogen, we find only a small number of women are on the recommended form of oestradiol, and an even smaller number use a recommended transdermal preparation. Most TS patients are receiving Ethinylestradiol which is a synthetic form, this is an issue that requires attention for both existing and future transitioning women. More recently the paediatric team has moved to using a physiological oestrogen with preference to transdermal route. Going forward this information should be shared with the adult team during transition to ensure appropriate HRT is used. Additionally, a proportion of women in our cohort are inappropriately maintained on oestrogen only HRT or without any prescribed HRT, with no evident reason identified for this from their clinical record. These women were all noted to be lost to long-term follow-up. Single-form oestrogen HRT in females increases the risk of endometrial cancer, therefore progesterone is routinely prescribed with oestrogen to counteract the proliferative effects on the endometrium (15)

We believe that various factors may explain why a substantial number of girls are lost to endocrine follow-up at our centre. The median age of transfer in our clinic at 18 years likely coincides with social and educational transitions. In addition to leaving the paediatric TS clinic, girls are likely to be making other life choices regarding further education and career which may necessitate leaving the family home (16). Attending an adult clinic may not take priority over these changes particularly if the opportunity to discuss ongoing health monitoring, beyond growth and puberty, has not been met whilst these girls are either in the paediatric or transition TS clinic (17).

This review of our service has highlighted that the current model of transition is unsuccessful in establishing and maintaining attendance in an adult endocrine clinic and therefore unsuccessful at providing the necessary monitoring and intervention to maintain health in women with TS beyond paediatric services. Changes to our current transition model could include using a transition coordinator, whose role would be to provide important information regarding health to young women with TS, sending reminders regarding upcoming appointments and remaining a point of contact until young women are in established adult endocrine follow-up. In addition, involving families and TS patients to address their specific concerns and providing them with written information and guidance prior to transition could improve retention. The services in the West of Scotland will introduce dedicated TS clinic in the adult setting with paediatricians attending multiple visits to remain a visible contact and link. It has previously been shown that the combination of a structured approach with an official transition coordinator and attendance of the paediatrician at a first adult clinic appointment led to a more efficient transition process and was preferred by families (18).

## Data availability statement

The original contributions presented in the study are included in the article/supplementary material. Further inquiries can be directed to the corresponding author.

## Author contributions

Authors BZ, AM conceived the idea, the script was written by BZ and received editorial input from AM and MD. AS, MF contributed to script idea as well as providing relevant patient information. All authors contributed to the article and approved the submitted version.
